# Workflow analysis and evaluation of a next-generation phenotyping tool: A qualitative study of Face2Gene

**DOI:** 10.1038/s41431-025-01875-0

**Published:** 2025-05-23

**Authors:** Katharina Wenderott, Jim Krups, Fiona Zaruchas, Peter Krawitz, Matthias Weigl, Hellen Lesmann

**Affiliations:** 1 Institute for Patient Safety, University Hospital Bonn, Bonn, Germany; 2 Institute for Genomic Statistics and Bioinformatics, University of Bonn, Bonn, Germany; 3 Institute for Human Genetics, University Hospital Bonn, Bonn, Germany

**Keywords:** Genetic counselling, Genetic counselling, Genetics research, Diagnosis, Paediatrics

## Abstract

The diagnosis of rare genetic disorders often involves prolonged delays, with facial features serving as key diagnostic clues. Next-Generation Phenotyping (NGP) tools, such as Face2Gene, utilize Artificial Intelligence (AI)-driven algorithms to analyze patient photographs and list differential diagnoses based on facial dysmorphism. Despite their growing use and proven clinical value, their integration into clinical workflows remains poorly understood. This study evaluates Face2Gene’s implementation into routine clinical care with barriers and facilitators to successful adoption. We conducted a literature review, followed by in-depth interviews with 15 geneticists across university hospitals in Germany. Results showed an overall positive appraisal of the tool among clinicians with emphasis on its usability. Key workflow barriers comprised IT integration and patient consent process. Despite being an additional step in the diagnostic pathway, Face2Gene has been effectively incorporated into geneticists’ diagnostic routines, facilitating decision processes, and potentially expediting diagnoses for some patients. Our findings contribute to the existing literature on NGP technologies by demonstrating that effective integration of Face2Gene can enhance clinicians’ efficiency and quality of work. To maximize impact of NGP technologies in genetic medicine, future implementation efforts should strive for clinicians’ acceptance particularly through user-friendly design and sustained organizational support in course of workflow implementation. Study registration: German Register for Clinical Trials (DRKS) DRKS00032436

## Background

Despite ongoing advances in sequencing technologies, diagnosing rare genetic disorders remains a lengthy and complex process, often leading to a prolonged diagnostic odyssey for affected individuals. On average, the time to diagnosis is 4–5 years, with some cases extending beyond a decade [1]. A key element in achieving a reliable diagnosis is often the phenotype of the patient. Since approximately 40% of rare diseases are associated with facial abnormalities, facial features can provide valuable diagnostic clues, particularly in disorders linked to facial dysmorphism [2, 3].

Alongside significant advancements in Next-Generation Sequencing, a growing array of Next-Generation Phenotyping (NGP) algorithms has emerged that are mostly based on recent advancements in computer vision and Artificial Intelligence (AI). The integration of such AI algorithms into more complex user interfaces results in NGP tools that are used in clinical practice to analyze phenotypes and suggest differential diagnoses based on facial images of patients [4–7]. The primary goal of these tools is to streamline the diagnostic process and reduce the time to diagnosis. Numerous studies have already demonstrated the technical validity and clinical utility of NGP by assessing its performance [8–10]. A recent large-scale study in Germany reported a significant improvement in the prioritization of exome data by using AI-based results of image analysis (PEDIA approach) [11, 12]. Face2Gene (F2G) is a software suite that provides access to NGP algorithms, namely DeepGestalt and GestaltMatcher, and is widely used by clinicians for analyzing patient photographs. F2G is also already implemented in clinical routine care in some clinics for human genetics [6, 7, 13].

Despite the growing use of NGP tools such as F2G in clinical practice, systematic investigations are lacking on how these tools are integrated into everyday clinical workflows or how efficiently they are utilized in routine care. Additionally, the factors influencing clinicians’ acceptance of these technologies have not been thoroughly assessed. Understanding these challenges is crucial to evaluating the real-world impact of NGP tools, as their true value depends on their acceptance, effective integration, and consistent use in clinical settings [14]. The work system model developed by Carayon [15, 16] provides a useful framework to study the implementation of novel technologies such as NGP tools. This sociotechnical model conceives workflows as an interaction between five key elements: people, tasks, tools and technologies, physical environment, and organizational structures [15]. Introducing a new technology, such as an AI-driven or NGP tool, affects all elements of clinical work systems altering the relationships between these elements [17]. By adopting this systemic approach, the model captures significant changes in the work system as well as the impact on human interaction with the technology [14, 18, 19].

This study aims to conduct a provider-focused evaluation of the integration of NGP tools into clinical workflows, using F2G as a use case. Furthermore, it seeks to identify the barriers and facilitators to adopting NGP tools across different clinical settings, offering insights to support their broader implementation in the future.

## Materials and methods

### Study design

In a multi-stage study design, we first conducted a literature review assessing studies which applied F2G, which was followed by a cross-sectional interview study. The study was pre-registered (DRKS00032436) and approved by the Ethics Committee of the Medical Faculty, Bonn University (2023-161-BO). The reporting of this study adheres to the COREQ checklist (COnsolidated criteria for REporting Qualitative research) [20].

### Use case: Face2Gene

Face2Gene (https://www.face2gene.com/; FDNA, Atlanta, GA) uses a deep convolutional neural network called DeepGestalt to detect syndromic phenotypes in facial photos [6]. DeepGestalt classifies 300 syndromes, with a 90% accuracy rate for including the correct diagnosis among the top 10 suggestions. F2G is offered as a web service accessible for healthcare providers and is currently used in 130 countries across 2.000 clinics according to the manufacturer FDNA [21].

### Literature review

To provide a better foundation and contextualization of our results a literature review was conducted using ‘Face2Gene’ as a search term in the databases PubMed, Embase, Web of Science, and CENTRAL. The search was finalized on May 2^nd^ 2024. Publications were de-duplicated in Rayyan and screened in Zotero. Inclusion decisions adhered to the PICOS framework and were conducted by one researcher (KW) (see Appendix 1). After screening the full-texts of studies matching the selection criteria, one researcher (KW) extracted the study design, condition, case/s, controls, population, F2G application used and outcomes assessed in regard to F2G into MS Excel. The extracted data were reviewed by a second researcher (JK).

### Interview study with clinicians

#### Participants

A convenience sampling approach was applied. In Germany, F2G has already been widely adopted within clinical genetic departments and, to a growing extent, in pediatric settings. Clinicians from pediatric and genetics departments in German university hospitals were invited, with an expected sample size of 30. Licensed physicians in human genetics or pediatrics with B2-level German language proficiency were included. Excluded were professionals with no experience with F2G.

#### Measures and content

The semi-structured interview guide (Appendix 2) was adapted from a German study on AI integration [22]. During the study development, the research team met and discussed relevant aspects for the interview guideline and adapted it to the specific context. Following its development, the interview guide underwent preliminary testing with a team member to assess its comprehensibility and clarity. The interviews lasted approximately 30 min. After mutual introductions, participants were asked about their age, gender, clinic, department, job title, and their professional experience.

For the assessment of attitudes towards AI, participants were asked one open question on general expectations regarding AI in their medical discipline. Additional questions addressed chances, risks, and perceived effects of AI-based technology use on patient safety. If necessary, interviewees were asked to elaborate their answers. Additionally, they were asked if their appraisal on AI changed since they started working with an AI tool.

Regarding use and usability, participants were asked about their perceived usefulness, perceived ease of use, and actual use of F2G. In terms of workflow integration, the questions primarily focused on how F2G was incorporated into clinical routines. We explored factors that influenced the decision to use or not use the AI tool before its application, as well as conditions that facilitated or hindered its use after the decision was made, i.e., during its integration into the workflow. Facilitators were defined as “any factor that promotes or enhances the integration or use of the AI system in the workflow,” while barriers were described as “any factor that limits or restricts the integration or use of the AI system” [23].

#### Procedure

Departments were contacted via email with a study description and Unipark survey link to schedule the interview and obtain informed consent. Interviews were conducted either in person or via telephone, for both settings it was ensured that the interviews took place in a separate room to minimize disturbances. For compensation, participants received a 60€ incentive. Semi-structured interviews in German were audio-recorded and conducted by graduate assistants with a background in medicine (FZ) and psychology (JK), after training by lead researcher KW. Demographic data was collected separately on paper (Appendix 3). Data saturation was indicated by no novel ideas emerging from the interviews and repetitions occurring across interviews and statements.

#### Analysis

Interviews were transcribed using audio.whisper in RStudio [24]. Data extraction on the use of F2G which were usage, frequency of use, purpose and medium was done by KW and checked by JK.

Next, we used qualitative content analysis following the method of Kuckartz and Rädiker [25] to identify barriers and facilitators clinicians experienced when using F2G. To guide this process, we created a codebook, which is a set of instructions defining the goals of the analysis, the structure, and the meaning of different codes. Two researchers (KW, JK) independently coded the transcripts using MAXQDA 24, resolving discrepancies through discussion or by consulting a third researcher. The list of main categories and their definitions can be found in Appendix 4. In the next step, we created subcategories using an inductive approach. We compiled these categories into a detailed codebook with clear definitions. To ensure consistency among raters, we tested the codebook in three interviews, discussed any coding discrepancies, and refined the definitions as necessary. Additionally, authors JK and KW individually identified the work system elements relating to the dimensions of facilitators and barriers, establishing a consensus through discussion; consistent to Wooldridge et al. [26, 27] and Wenderott et al. [14].

## Results

### Literature review

**Fig. 1:**
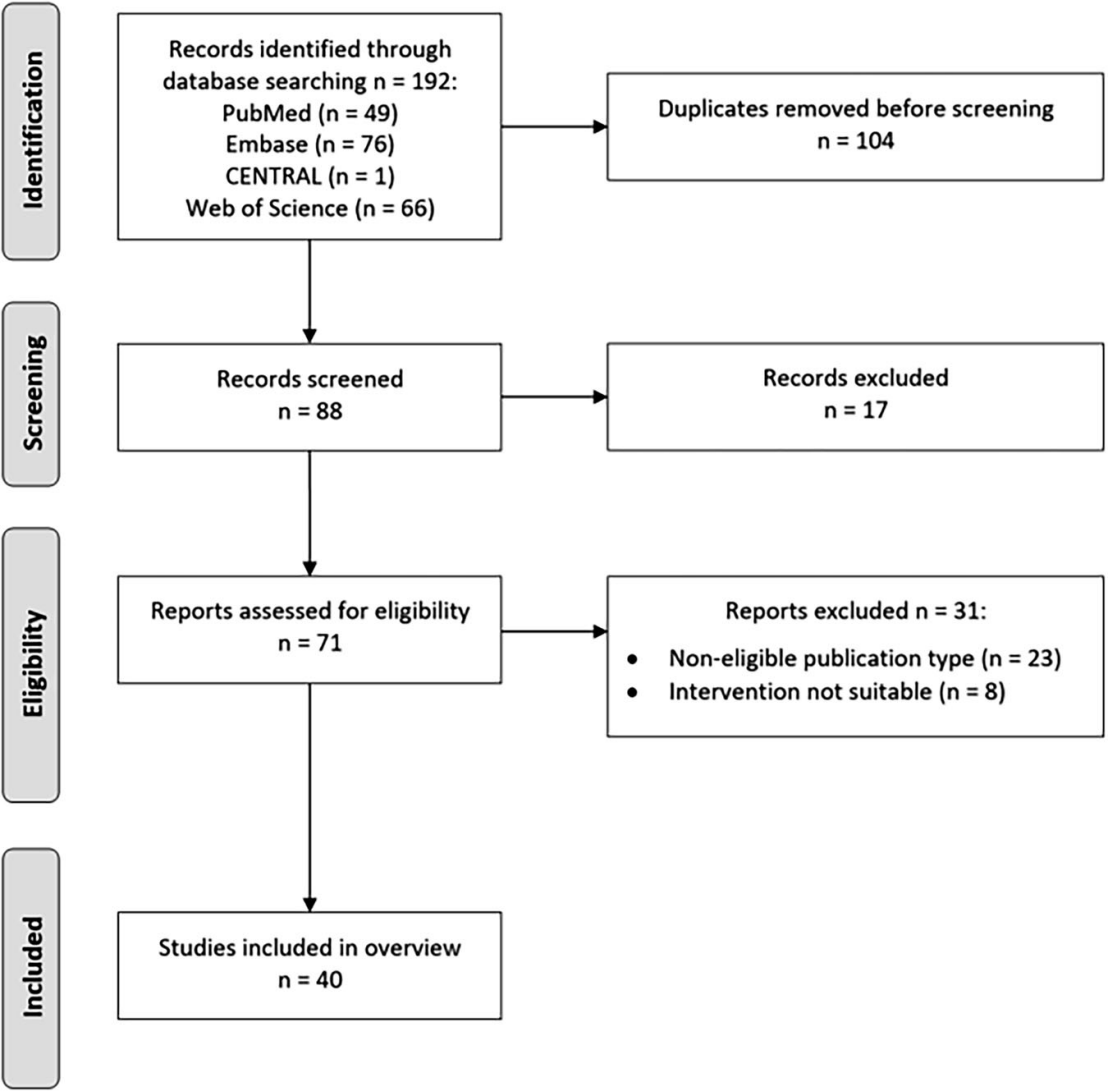
Literature review flow chart.

The search yielded 192 publications, of which 40 were included after screening (see Fig. 1), a list of excluded studies is provided in Appendix 5. Appendix 6 lists key characteristics, populations, and outcomes of the included studies. Included studies comprised 11 case-control, 11 retrospective validations, 10 single-case, 5 multi-case, and 2 prospective studies. One study did not clearly describe the study setup [10].

Only one study, Marwaha et al. [13], assessed clinicians’ experiences with F2G, while the vast majority of studies focused on case descriptions or accuracy. Marwaha et al. [13] used a six-question survey with ten respondents to evaluate F2G’s use and usefulness one year after implementation. They also assessed its use as an educational tool for trainees. Overall, usability was rated positively, though participants expressed concerns about its usefulness, informed consent, and data security. To summarize, our literature review showed that the literature concerning systematic evaluations of clinicians’ experiences is missing, while F2G has been integrated into routine use and demonstrated a good accuracy.

### Clinicians’ interviews

#### Sample

Seventeen interviews were conducted between October 2023 and April 2024, with two excluded due to no contact with F2G, leaving a final sample of 15 clinicians (see Table 1). Participants from six German cities were affiliated with human genetics departments at university hospitals. One was also a pediatric specialist. Mean age was 40.07 years (Standard deviation [SD] = 10.33), with 11 females and 4 males.

**Table 1:** Details on interviewees.

No.	Position	Work experience (in years)	Use	User Group	Purpose for use	Medium	Reported frequency of tool use
2	Attending	14	No	Non-User	Patient care	Computer	10 times
4	Resident	1	Yes	Routine	Patient care	Computer	Almost daily, 1–2 times per day
5	Resident	5	Yes	Occasionally	Research, patient care	Smartphone	1–2 cases & a research project
6	Resident	2	Yes	Routine	Patient care, research	Computer	All patients with dysmorphic features
7	Attending	22	Yes	Routine	Patient care	Computer	Half of patients
8	Attending	19	Yes	Occasionally	Patient care	Computer	Two times a month
9	Resident	2	Yes	Routine	Patient care, research	Computer	All patients with dysmorphic features
10	Attending	25	No	Non-User	Patient care	No information	No use
11	Resident	2	Yes	Routine	Patient care	Computer	Once a week
12	Attending	23	Yes	Occasionally	Patient care	Computer	Every third patient (30–40 times a year)
13	Attending	14	Yes	Routine	Patient care	Computer	All patients with suspected genetic syndrome
14	Attending	21	Yes	Routine	Patient care	Computer	Twice a month
15	Attending	15	Yes	Routine	Patient care, research	Computer	All patients
16	Attending	15	Yes	Routine	Patient care	Computer	Almost all patients with dysmorphic syndrome
17	Resident	0.8	Yes	Routine	Patient care	Computer	All children

The interview duration was on average 22:08 min (SD = 06:16 min). Ten participants used F2G in their routine workflow, three used it occasionally, and two used it before but did not continue. These experience- and usage-levels were used to distinguish between the three user groups: (1) Non-Users, who had tried F2G but did not continue to use it; (2) Occasional Users, clinicians who used it only occasionally for specific cases; (3) Routine Users, who used it repeatedly for almost all their eligible patients.

#### Clinicians’ reports on AI use in genetics

When participants were asked about their expectations regarding the use of AI in their field, they most frequently described several potential benefits: supporting clinical care processes (10/15; 10  out of 15 interviewees), enhancing efficiency (8/15), improving diagnostics (7/15), and easing the burden on healthcare professionals (4/15). However, they also expressed concerns about various risks associated with AI use, such as the potential for misuse (9/15), overtrust (8/15), bias in AI solutions (3/15), and the possible loss of professional skills (2/15). Details can be found in Appendix 7 and 8. Concerning factors that would encourage their acceptance of AI solutions, participants most often emphasized the importance of data protection (10/15), particularly when handling medical data. Transparency (9/15) was also a key factor, with one participant stating, “I definitely want to know how something works when I upload patient data there. Even if I can’t always fully understand everything, I think such transparency greatly helps in the acceptance of these tools” (ID: IV08). Additionally, the source of the AI solution (6/15) and the data used to train the AI (6/15) were significant considerations. As one participant noted, “Those are definitely things I would question, just like whether a university hospital is behind it or if it’s a commercial provider. And who is financing these commercial providers or what kind of organization is behind them” (IV09).

#### Evaluation of Face2Gene

Drawing upon the clinicians’ reports of F2G use, we identified the following F2G workflow. It starts with patient consultation, where clinicians obtain consent from patients or guardians and take photographs. A separate informed consent is required for uploading these images to F2G for processing and providing diagnostic suggestions for report generation.

**Fig. 2:**
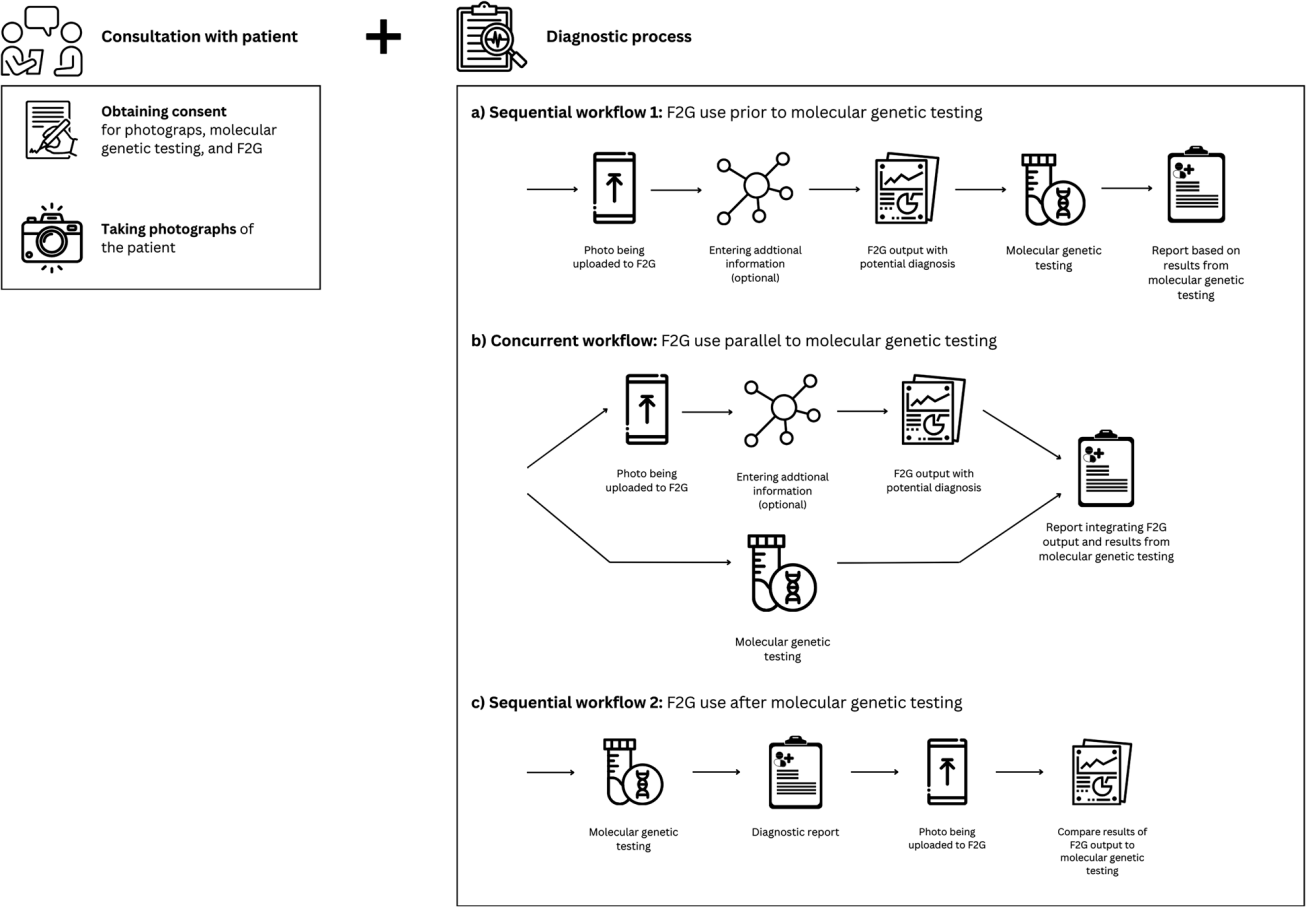
Prototypical workflows for using F2G after the patient consultation.

Only two participants used F2G during patient consultations. For using F2G after the patient contact, interviewees described three distinct workflows: nine clinicians used it sequentially before molecular genetic testing for initial diagnostic impressions; four applied it concurrently and combined the results from both methods when generating the diagnostic report; and three sometimes used it afterwards to describe cases or evaluate molecular results. These workflows are illustrated in Fig. 2.

Participants reported to switch between different workflows, as their actual decision whether and when they use F2G in routine patient care was influenced by various factors. Most interviewees (12/15) noted that time pressure significantly affected its use, while 11 mentioned that patient characteristics influenced their decision to use. Organizational factors, including workspace, digitalization level, and overall acceptance were also important, and three participants indicated that F2G’s reputation in the professional community impacted their usage decisions. For detailed definitions and examples, refer to Table 2.

**Table 2:** Reported factors influencing clinicians’ decision to use Face2Gene.

Factor	Definition	Number of Statements	Number of Interviews	Example Statement
Time pressure	Workload and time pressures in the department influencing the use of F2G.	14	12	“I think if there is time pressure, I would use Face to Gene […] less than I normally would.” (IV 09)
Patient characteristics	Patient characteristics which influence the decision to use, e.g. highly dysmorphic facial features of a patient.	12	11	“So, if I have a patient who looks noticeably dysmorphic for me, then I definitely want to use it.” (IV 15)
Organizational factors	Hospital organization providing support, technical equipment or a positive attitude towards the use of F2G.	10	6	“[…] if I had a professional smartphone, I think I would use it more likely.” (IV 05)
Popularity of F2G	The prominence and/or reputations of F2G having an impact on F2G usage.	4	3	“[…] you simply hear from other colleagues at congresses that they also use it and that it has also contributed to solving the disease in question.” (IV 13)
Trustworthiness of the source of F2G	Trustworthiness attributed toward the source and/or developers of F2G impacting the decision to use F2G.	2	1	“[…] many colleagues […] don’t get involved with projects like F2G for data protection reasons, because they say Face2Gene […], they have their servers in the USA […]” (IV 06)

Twelve of the 15 participants reported positive experiences with F2G. One noted, “Yes, F2G is very useful, […] We would never make a diagnosis based on this alone without molecular correlation, but as a decision-making aid, it’s definitely a good one” (IV07). In contrast, three participants shared negative experiences, including a non-user (IV02) who said, “I used it in its very early stages, and it didn’t really help me. I was more frustrated. […]”. Participants also indicated that the tool’s usefulness varied based on factors like the specific patient (6/15), purpose of use (4/15), and the clinician’s experience (3/15). One participant (IV06) remarked that “the usefulness is very high, especially at the beginning of one’s career. […] Once you gain experience, you might be able to figure things out on your own. I often see this with experienced colleagues—they already have their own ideas, which Face2Gene might then support”.

#### Facilitators and barriers of using Face2Gene

A total of 87 statements was collected on facilitators and barriers for successfully integrating F2G into clinical work. Facilitators were mentioned more frequently (51/87 statements, 15/15 interviews) than barriers (36/87 statements, 12/15 interviews). These were categorized into eight dimensions, with definitions provided in Table 3. When examining the identified dimensions of workflow integration, usability, time investment, and accessibility emerged as the dimensions with the highest number of facilitators. These facilitators were largely related to the AI solution itself. Two dimensions—obtaining consent and additional work steps—were identified solely as barriers and related to tasks required of the users. Mixed evaluations were reported regarding local support and IT integration, which varied across organizations.

**Table 3:** Definitions, frequency, and exemplary statements of facilitators and barriers for Face2Gene implementation.

Dimension	Definition	Work System Elements						Facilitators			Barriers		
		P	T	T/T	O	PE	EE	No. of Statements	No. of IVs	Example	No. of Statements	No. of IVs	Example
Usability	Users can interact effectively and intuitively with F2G to accomplish their goals.	x		x				34	15	“So, I think it’s a very clear program, you can very easily create new cases and simply upload the image by drag & drop […]” (IV 04)	8	5	“When you’ve uploaded the picture, where you then enter the features, so it’s somehow not so clearly separated, so every time I stumble over it again […]” (IV 14)
Time investment	The amount of time needed to integrate and use F2G in the daily clinical workflow.		x	x				6	3	“So, it’s also something you can use very quickly. It’s not particularly time-consuming.” (IV 07)	5	4	“You are dependent on the photos, which can take a bit of time, that you can’t do it straight after the consultation [..]” (IV 11)
Obtaining consent	The process of getting the patients’/legal guardians’ consent is affecting the use of F2G.	x	x		x			0	0	NA	9	4	“And all the paperwork, maybe you forgot to get the declaration of consent. You then have to obtain it afterwards.” (IV 06)
Additional work step	Using F2G is an additional work step in the process.		x	x				0	0	NA	6	5	“Well, it’s just another chunk of work, I say, that you don’t necessarily have to do to get a diagnosis most of the time.” (IV 02)
Local IT integration	Ensures that F2G can seamlessly communicate and share data with other technologies used.			x	x			1	1	“And the sending of patient photos via email works quite well for us, as we have a secure cloud for that.” (IV 05)	6	6	“So that was not integrated and is not integrated yet. So, we have to download the images manually and then upload them back into Face2Gene. […]” (IV 02)
Accessibility	The accessibility of F2G, e.g. being accessible on multiple devices, influences its workflow integration.			x	x			5	5	“It fits in well, because you can do it whenever you want, even on your PC, and you can theoretically log in anywhere, no matter which device you’re working on […]” (IV 04)	0	0	NA
Local support	At the institution clinicians have support when using F2G.	x	x		x			3	3	“There is support for it and some use it more, some use it less, but it is generally accepted […].” (IV 14)	1	1	“At the moment, it’s more the case that only a few people even know about the app.” (IV 05)
Influence by direct supervisor	The supervisor or head of the team is being supportive or unsupportive for using F2G.	x			x			2	2	“[…] my boss, who keeps suggesting that we should do it […]“ (IV 02).	1	1	“And I definitely notice that not everyone is equally enthusiastic about these things and this influences people’s use and sometimes also their interpretation.” (IV 06)

*No* number, *IV* interview, *P* people, *T* tasks, *T/T* tools and technologies, *O* organization, *PE* physical environment, *EE* external environment, *F2G* Face2Gene, *IT* Information technology, *NA* not applicable.

## Discussion

Artificial Intelligence in genetic medicine offers great potential for improving diagnosis and treatment. However, sustainable AI implementation in clinical settings presents challenges for providers and organizations. This study evaluates the use of Next-Generation Phenotyping tools in the diagnostic process, focusing on F2G. Our review revealed only one study addressing F2G user evaluations [13]. This lack of including healthcare provider viewpoints in AI integration is not unique to genetics, also appearing in other medical fields [28–30]. Our research contributes critical factors influencing the adoption and rejection of NGP tools and elucidates the conditions that either facilitate or hinder their utilization within clinical workflows in genetics.

Our review highlighted that F2G has successfully mastered the translation from usage in a research context into routine care, a critical step for AI tools in healthcare [29]. Although numerous studies utilizing F2G to report case descriptions, they frequently fail to include specific workflow details. The predominant focus on performance metrics, such as the accuracies of DeepGestalt or GestaltMatcher, often neglects clinicians’ experiences, which is essential for the successful adoption of novel technologies [31, 32]. Marwaha et al. employed a brief questionnaire to capture user opinions one-year post-implementation at a single institution, providing an initial overview of user experiences [13]. Building on this, our study employed a semi-structured interview methodology with participants from diverse hospitals to enhance these findings. Although Marwaha et al. found F2G to be beneficial for diagnostic decision-making, they also identified significant reservations concerning accuracy, informed consent, confidentiality, and patient uptake [13]. In alignment with these findings, our participants reported favorable usability of F2G; however, they expressed concerns regarding the informed consent process.

Our interviewees highlighted that the use of F2G is just one component of the diagnostic process. Moreover, all users reported using it in addition to molecular genetic analyses. The extracted workflows align with research on integrating AI into work processes [28]. Interviewees described F2G as easy to use and evaluating its usefulness positively, which are key contributors for the intention to use of a novel technology [33]. Additionally, we identified several key factors impacting the decision to actually use F2G. Time pressure was the most frequently cited factor influencing participants’ use of F2G, aligning with findings from other AI integration studies [18, 22]. However, time pressure was noted as less intense in genetics than in other specialties like pediatrics. Most users reported utilizing F2G for dysmorphic patients, while some did so out of curiosity or when a suspected condition was unclear. This is noteworthy, as the performance of these tools relies on the distinctiveness of facial dysmorphisms [7]. To optimize the cost-benefit ratio of time and AI performance, participants reported to select suitable patients for facial analysis, as indicated by their usage patterns. For most users, specific patient characteristics were central to their decision to use F2G, as reflected in their responses about how frequently they utilize the tool. Given the high proportion of diseases with facial dysmorphism [2, 3], NGP tools will continue to have ample use cases. In a scientific context, however, the analysis of less distinct disorders could also be useful, as AI can also recognize patterns that are not always apparent to humans [34].

An analysis of the workflow revealed that the consultation and patient photography are standard steps of genetic assessments. This may account for the observed absence of specific facilitators or barriers linked to F2G in this stage. However, from obtaining patient consent onward, additional implementation factors for successful F2G integration were identified. We observed more facilitators than barriers, potentially indicating a good workflow fit. The distribution of facilitators and barriers across different elements of the work system model highlights the complexity of adopting AI tools like F2G. Facilitators such as usability, time investment, and accessibility are closely tied to the AI solution itself namely the element of tools and technology, emphasizing the importance of good design when implementing AI in clinical settings. In contrast, barriers like obtaining consent and additional work steps are linked to the element of tasks, suggesting that administrative processes may hinder smooth integration. Additionally, the lack of work-issued smartphones or tablets for many participants presents a significant obstacle, forcing a reliance on external photographers and desktop computers, which complicates the workflow. These issues point to broader organizational challenges, such as the need for better local support and more seamless IT integration, which were met with varying levels of satisfaction among participants. The amount of barriers and facilitators associated with the element organization highlights the impact organizational support could have, especially as it was also among the factors the inform users’ decision to use F2G. For example, the process of obtaining consent was a central barrier to use, which could be mitigated by the organization providing standardized forms for using F2G. It is crucial to clarify that patient consent is only required for research-related activities, such as the technical validation of the AI algorithm’s performance. Similar to numerous Next-Generation Sequencing (NGS) platforms, NGP software is frequently designated ‘for research purposes only’, which may lead to user confusion. If these tools are approved as medical devices for decision support in the future and become part of accredited workflows, this usage would typically not necessitate additional patient consent. The work system element ‘people’ was identified mostly for task-related facilitators and barriers, but three users also identified their supervisor as having a strong impact on their workflow using F2G. We identified no facilitators or barriers related to the environment, likely because the introduction of F2G as a web-based AI solution did not lead to any changes.

Salwei and Carayon outlined three essential sociotechnical considerations for integrating AI into healthcare systems: the alignment with work systems, compatibility with existing workflows, and enhancement of clinical decision-making processes [17]. Through analyzing the work system barriers and facilitators, our study was able to demonstrate an overall good fit of F2G in the work system, supporting their first consideration. In examining how F2G fits into the workflow and the positive feedback regarding the time required to use it, we found that it integrates well with the existing workflow, which supports the second consideration. Although F2G is not a mandatory step in the diagnostic process, participants found it valuable for diagnostic purposes and case discussions, which aligns with findings from Marwaha et al. [13]. This supports the third consideration regarding workflow integration. Overall, we found that F2G is a well-integrated AI solution, yielding positive outcomes such as enhanced user satisfaction, shorter time to diagnosis, and greater acceptance of the technology. Clinicians’ positive appraisal regarding the use of F2G corresponds with the primary advantages participants sought from AI in genetics, namely improved diagnostics and increased efficiency, which are relevant across healthcare setting [35, 36]. The information gained from this study can be used to successfully implement other NGP tools such as GestaltMatcher and Phenoscore [4, 7]. Even though the scientific benefit of these tools has been already proven by demonstrating their accuracy, their seamless transfer into clinical routine is essential to actually promote significant improvements in diagnoses of rare genetic disorders. This can only be achieved by identifying and addressing concerns of clinicians during the implementation ensuring a smooth fit into the workflow.

Participants generally viewed AI and NGP tools as beneficial for improving care processes, efficiency, diagnostics, and reducing healthcare workloads. However, they expressed concerns about potential misuse, over-reliance, bias, and loss of professional skills. This resonates with a study by Hallowell et al. which explored stakeholder perspectives on NGP technology [37]. They additionally highlighted that NGP tools could democratize access to diagnoses—an aspect not raised in our interviews, likely due to the widespread availability of genotyping methods in Germany. This shows a limitation of our study: all participants were recruited within Germany to ensure comparable workflows. Given that F2G is deployed globally, future studies should examine workflow integration across different countries and healthcare settings to identify universally applicable recommendations. Additionally, our literature review featured a rapid synthesis of available studies involving F2G, whereas a more systematic and rigorous approach might have been more effective in ensuring that all relevant studies were included (i.e., from gray literature). Although our response rate was lower than expected, sufficient data saturation was achieved, as later interviews did not reveal new themes. It must be noted, that we aimed to include both pediatricians and geneticists; however, only geneticists responded. Pediatricians, who may benefit most from NGP for patients with facial dysmorphism, often have shorter visit times, making their perspective on the cost-benefit ratio of NGP particularly valuable [38, 39]. Future studies should prioritize this user group to assess their specific needs and challenges. Finally, the limited number of interviews, particularly with non-users, may have introduced bias. Future research should explore detailed comparisons between various user groups, possibly examining correlations with diverse user characteristics [40].

Our study enhances the existing literature on NGP technologies by evaluating the integration of F2G as a specific use case across various institutions. Through clinician interviews, we mapped the workflow of using F2G, which has been effectively incorporated into routine practice, despite being an additional step in the diagnostic process without replacing molecular genetic testing. This indicates that NGP technology can significantly improve healthcare efficiency and quality, provided that clinicians’ acceptance is further enhanced. Our analysis of facilitators and barriers using the work system model underscores essential considerations for future design and implementation, particularly the importance of user-friendly design and ensuring organizational support.

## Supplementary information

Supplementary Material

## Data Availability

The datasets generated during and/or analysed during the current study are not publicly available due to the sensitive nature of the interview content, which may compromise participant anonymity, but are available from the corresponding author on reasonable request.
