# Supplementary material for: Workflow analysis and evaluation of a next-generation phenotyping tool: A qualitative study of Face2Gene
Source: Eur J Hum Genet. 2025 May 23;33(10):1333–41. doi: 10.1038/s41431-025-01875-0 (PMC12480590; doi:10.1038/s41431-025-01875-0)
Supplement: Supplementary file 1 — Supplementary Material [file 41431_2025_1875_MOESM1_ESM.docx]

**APPENDIX**

# PICOS Statement Rapid Review

|  | Content |
| --- | --- |
| Population | Healthcare settings |
| Intervention | Face2Gene use |
| Comparison | NA |
| Outcome | Not model development or training |
| Study Type | Original studies |

# Interview Guideline

##
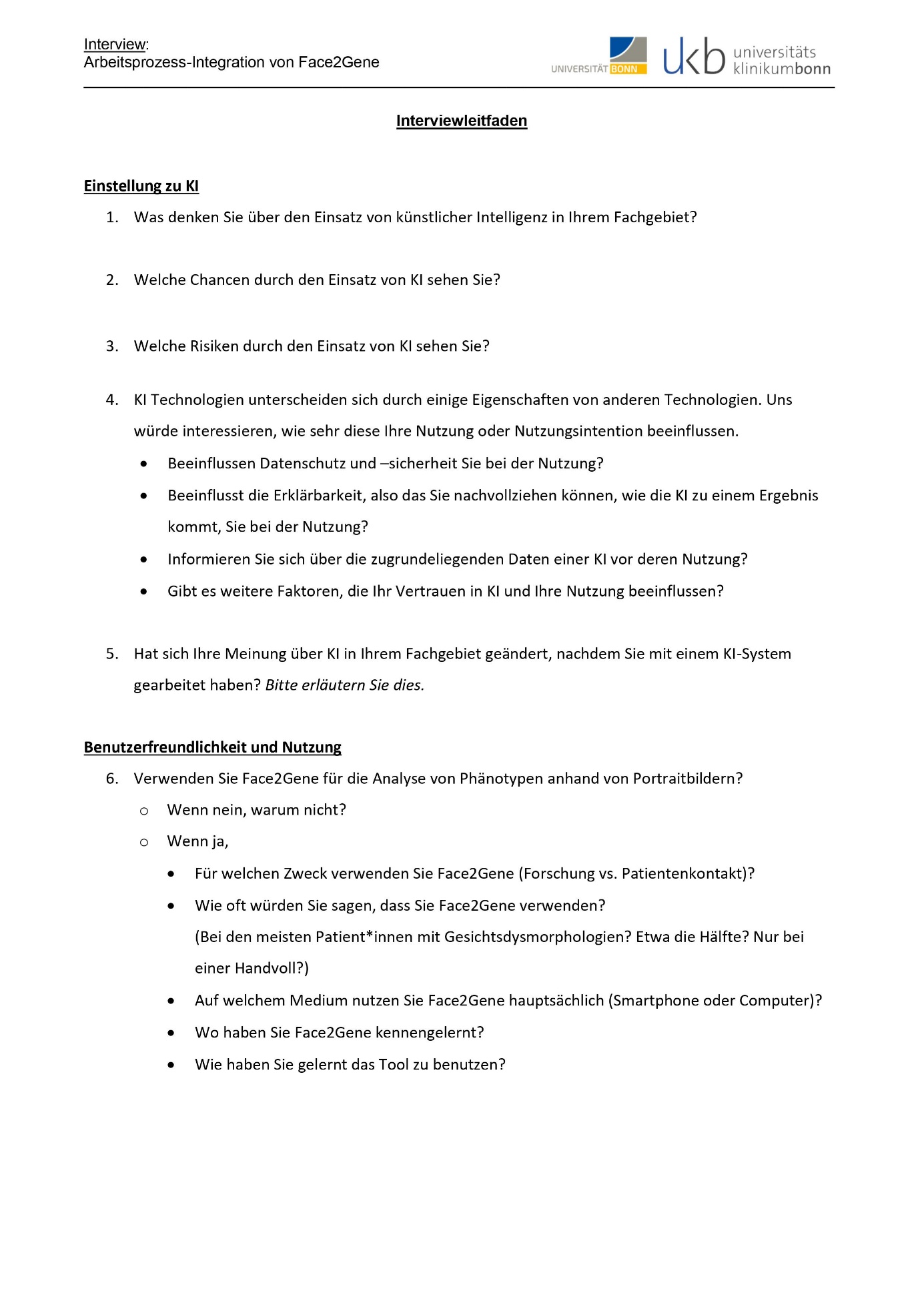
Original Version in German


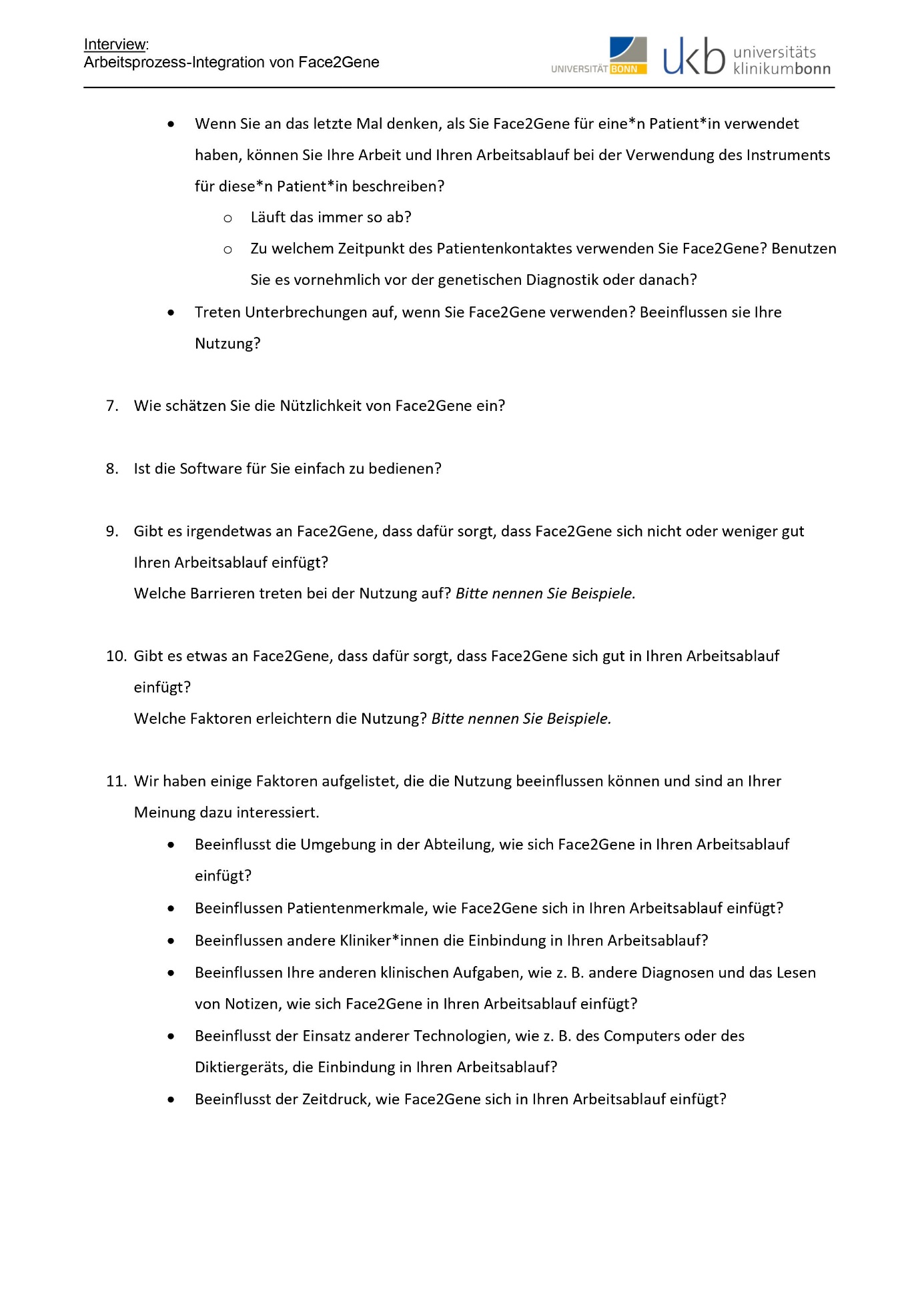

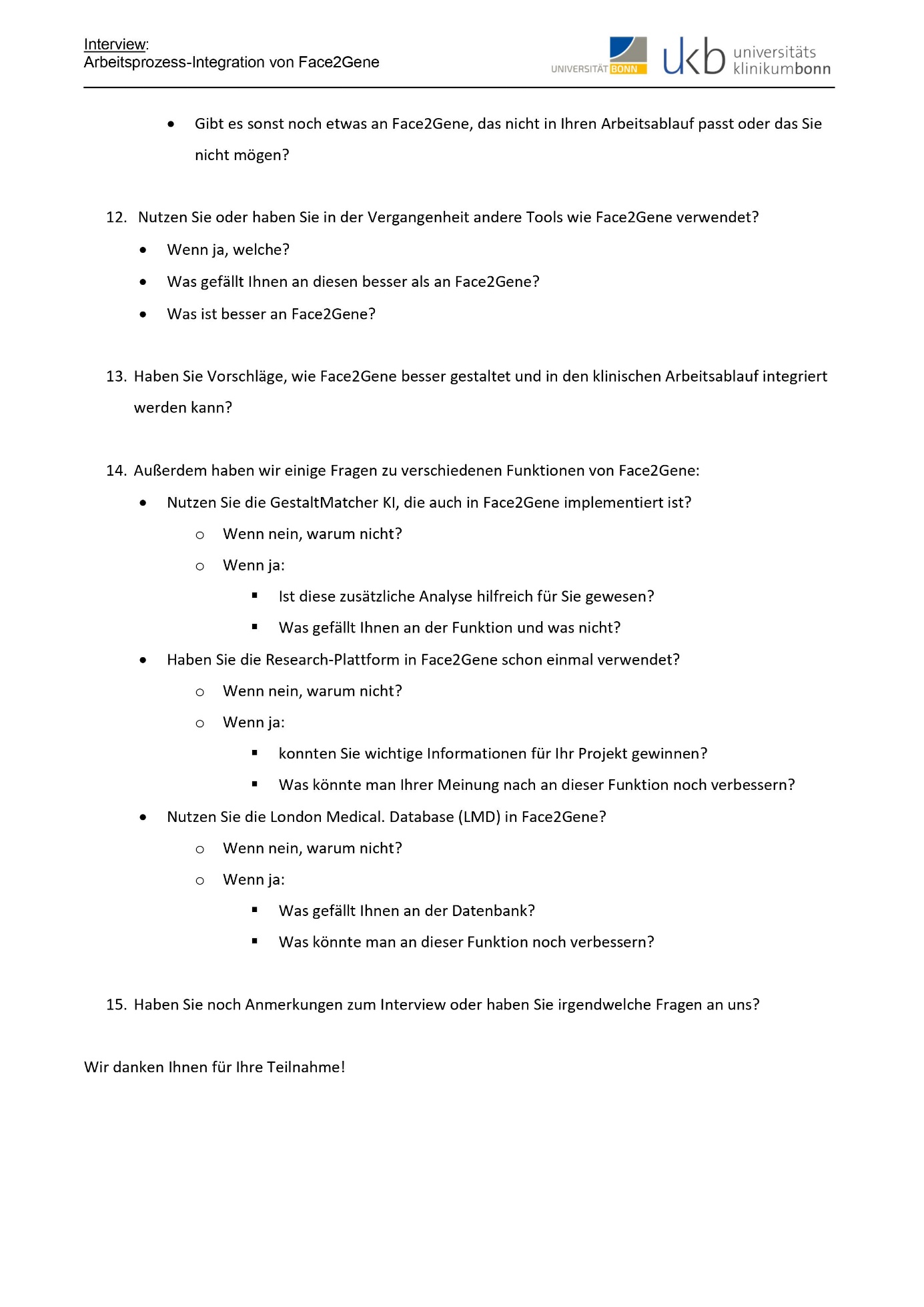


## Translated Version in English

**Interview Guide**

**Attitude towards AI**

1. What do you think about the use of artificial intelligence in your field?
2. What opportunities do you see from the use of AI?
3. What risks do you see from the use of AI?
4. AI technologies differ from other technologies in several ways. We would be interested in how much these factors influence your use or intention to use them.

- Do data protection and security influence your use?
- Does the explainability, i.e., being able to understand how the AI arrives at a result, influence your use?
- Do you inform yourself about the underlying data of an AI before using it?
- Are there any other factors that influence your trust in AI and your use of it?

1. Has your opinion about AI in your field changed after working with an AI system? Please elaborate.

**User-Friendliness and Usage**

6. Do you use Face2Gene for the analysis of phenotypes based on portrait images?

- If no, why not?
- If yes,
  - For what purpose do you use Face2Gene (research vs. patient contact)?
  - How often would you say you use Face2Gene? (For most patients with facial dysmorphologies? About half? Only for a handful?)
  - On what medium do you mainly use Face2Gene (smartphone or computer)?
  - Where did you learn about Face2Gene?
  - How did you learn to use the tool?
  - When you think about the last time you used Face2Gene for a patient, can you describe your work and workflow when using the tool for that patient?
    - Is it always like that?
    - At what point in the patient contact do you use Face2Gene? Do you primarily use it before or after genetic diagnostics?
  - Do interruptions occur when you use Face2Gene? Do they affect your use?

1. How do you assess the usefulness of Face2Gene?
2. Is the software easy for you to use?
3. Is there anything about Face2Gene that makes it not or less well fit into your workflow?

- What barriers arise when using it? Please provide examples.

1. Is there anything about Face2Gene that makes it fit well into your workflow?

- What factors facilitate its use? Please provide examples.

1. We have listed some factors that can influence usage and are interested in your opinion on them.

- Does the department environment influence how Face2Gene fits into your workflow?
- Do patient characteristics influence how Face2Gene fits into your workflow?
- Do other clinicians influence its integration into your workflow?
- Do your other clinical tasks, such as other diagnoses and reading notes, influence how Face2Gene fits into your workflow?
- Does the use of other technologies, such as computers or dictation devices, influence its integration into your workflow?
- Does time pressure influence how Face2Gene fits into your workflow?
- Is there anything else about Face2Gene that does not fit into your workflow or that you do not like?

1. Do you use or have you used other tools similar to Face2Gene?

- If yes, which ones?
- What do you like better about these compared to Face2Gene?
- What is better about Face2Gene?

1. Do you have suggestions on how Face2Gene can be better designed and integrated into the clinical workflow?
2. We also have some questions about various features of Face2Gene:

- Do you use the GestaltMatcher AI, which is also implemented in Face2Gene?
- If no, why not?
- If yes:
  - Has this additional analysis been helpful for you?
  - What do you like and dislike about this feature?
- Have you ever used the research platform in Face2Gene?
- If no, why not?
- If yes:
  - Could you obtain important information for your project?
  - What do you think could be improved about this feature?
- Do you use the London Medical Database (LMD) in Face2Gene?
- If no, why not?
- If yes:
  - What do you like about the database?
  - What could be improved about this feature?

1. Do you have any comments on the interview or any questions for us?

We thank you for your participation!

# DeMOGRAFIC DATA SHeet

## Original Version in German


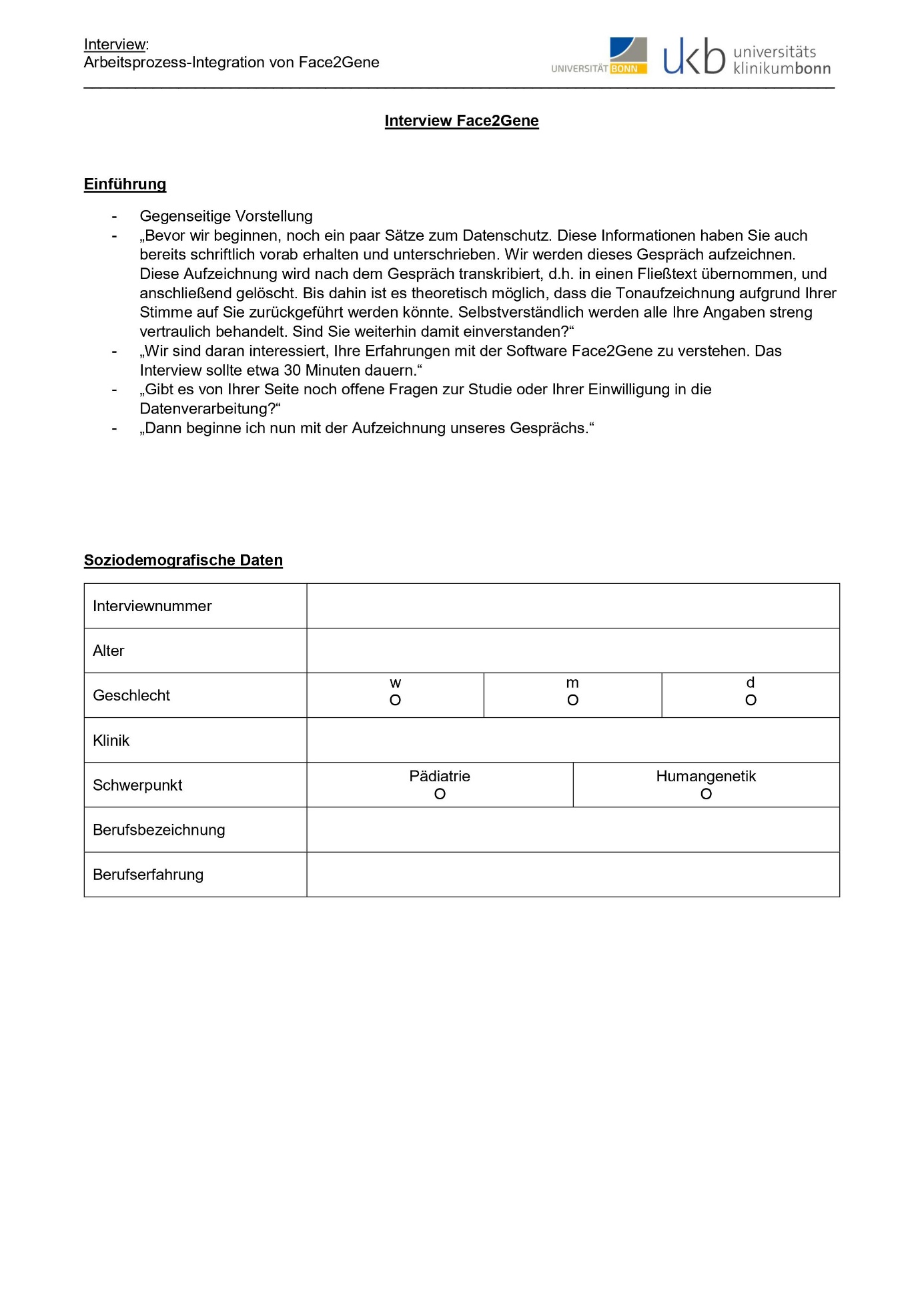


## Translated Version in English

**Interview Face2Gene**

**Introduction**

- Mutual introduction
- "Before we begin, a few sentences about data protection. You have already received and signed this information in writing in advance. We will record this conversation. This recording will be transcribed after the conversation, i.e., converted into continuous text, and then deleted. Until then, it is theoretically possible that the audio recording could be traced back to you due to your voice. Of course, all your information will be treated with strict confidentiality. Do you still agree?"
- "We are interested in understanding your experiences with the software Face2Gene. The interview should take about 30 minutes."
- "Do you have any further questions about the study or your consent to data processing?"
- "Then I will now start recording our conversation."

**Sociodemografic Data**

| Interviewnumber |  | | | |
| --- | --- | --- | --- | --- |
| Age |  | | | |
| Gender | w  Օ󠇊 | m  Օ󠇊 | | d  Օ󠇊 |
| Clinic |  | | | |
| Field of Specialisation | Pediatrics  Օ󠇊 | | Genetics  Օ󠇊 | |
| Job title |  | | | |
| Work experience |  | | | |

# Main Categories data extraction

| **Codes** | **Definition** |
| --- | --- |
| *AI in General* |  |
| AI Use | All conditions or descriptions of when and how participants would use an AI system.  e.g. „I need to know who developed the AI to decide whether I will use it.“ |
| Risks | All risks associated with the use of AI in healthcare in the future.  e.g. "Due to excessive reliance on AI, findings could be overlooked." |
| Chances | Includes all opportunities or expected positive changes through the use of AI in healthcare.  e.g. "I believe things will go faster because AI takes over tasks." |
| Barriers AI | Any factor that limits or restricts the integration or use of the AI system.  e.g. „The AI system does not work well with the local IT infrastructure, leading to long upload times.“ |
| Facilitators AI | Any factor that promotes or expands the integration or use of the AI system in the workflow.  e.g. „All my colleagues use the AI system, therefore I can ask them if I have any questions.“ |
| *Face2Gene* |  |
| Workflow | Description of the Workflow using Face2Gene  e.g. „First need to have permission from the parents, then we take pictures, which I then upload to the Face2Gene Platform.“ |
| External factors | External factors that are independent of the Face2Gene influencing its use.  e.g. „I as having multiple experiences with AI feel more confident using Face2Gene“ |
| Evaluation of Use | The evaluation of clinicians or patients of the usefulness of the Face2Gene.  e.g. „With the output from Face2Gene I am able to confirm my diagnosis most of the times.“ |
| Facilitators F2G | Any factor that promotes or expands the integration or use of Face2Gene in the workflow.  e.g. „It is very easy to use“ |
| Barriers F2G | Any factor that limits or restricts the integration or use of F2G  e.g. „Explaining patients who has access to the data is always complicated.“ |

# excluded Studies

| Authors | Year | Title | Journal | DOI | Reason for exclusion |
| --- | --- | --- | --- | --- | --- |
| Abdallah et al. | 2023 | The impact of artificial intelligence on optimizing diagnosis and treatment plans for rare genetic disorders | Cureus journal of medical science | 10.7759/cureus.46860 | Publication type |
| Agolini et al. | 2019 | Craniofacial appearance of a developmental disorder of chromatin remodeling in a girl with syndromic intellectual disability and a novel de novo microrearrangement encompassing HNRNPC gene | European journal of human genetics | 10.1038/s41431-019-0493-3 PT - Conference Abstract | Publication type |
| Anonymous. | 2019 | The 45th annual meeting of the american association for pediatric ophthalmology and strabismus | Journal of AAPOS | NI | Publication type |
| Bayat et al. | 2018 | PIGT-CDG, a disorder of glycosylphosphatidylinositol anchors: description of six novel patients and expansion of clinical characteristics | Epilepsia | 10.1111/epi.14612 PT - Conference Abstract | Publication type |
| Bezuidenhout et al. | 2020 | Mabry syndrome: A novel recurrent PGAP3 variant in an African population | European journal of human genetics | NI | Not found |
| Del Rocio Perez Baca et al. | 2023 | A novel neurodevelopmental syndrome caused by lossof- function of the Zinc Finger Homeobox 3 (ZFHX3) gene | European journal of human genetics | 10.1038/s41431-023-01337-5 PT - Conference Abstract | Publication type |
| Duong et al. | 2024 | Comparison of clinical geneticist and computer visual attention in assessing genetic conditions | PLoS genetics | 10.1371/journal.pgen.1011168 PT - Article | Publication type |
| Gripp et al. | 2021 | Syndromic disorders caused by gain-of-function variants in KCNH1, KCNK4, and KCNN3—a subgroup of K+ channelopathies | European journal of human genetics | 10.1038/s41431-021-00818-9 | Intervention |
| Gripp et al. | 2019 | Training a facial analysis software to recognize a very rare condition: Ayme-Gripp syndrome | European journal of human genetics | NI | Not found |
| Hennocq et al. | 2024 | Next generation phenotyping for diagnosis and phenotype-genotype correlations in Kabuki syndrome | Scientific reports | 10.1038/s41598-024-52691-3 | Intervention |
| Hsieh & Dukic et al. | 2019 | Exploring unknown genotype-phenotype relationship by nextgeneration phenotyping approaches | Medizinische genetik | 10.1007/s11825-019-0234-6 PT - Conference Abstract | Publication type |
| Hsieh & Hajjir et al. | 2019 | Exploring molecular interactions by clustering analysis of similarity scores from next-generation phenotyping approaches | European journal of human genetics | NI | Not found |
| Hsieh et al. | 2023 | Facilitating the molecular diagnosis of rare genetic disorders through facial phenotypic scores. | Current protocols | 10.1002/cpz1.906 | Intervention |
| Hsieh et al. | 2018 | Deep phenotyping for Deep Learning (DPDL): A searchable knowledge base which integrates genotype-phenotype information for clustering analysis in the phenotype space | Medizinische genetik | 10.1007/s11825-018-0176-4 PT - Conference Abstract | Publication type |
| Jang | 2017 | Application of facial dysmorphology analysis technology (Face2gene) in Korean rare genetic diseases | Annals of translational medicine | 10.21037/atm.2017.s052 PT - Conference Abstract | Publication type |
| Javitt et al. | 2022 | Evaluation of a computer-based facial dysmorphology analysis algorithm (Face2Gene) using standardized textbook photos | Eye | 10.1038/s41433-021-01563-5 | Intervention |
| Jiang | 2019 | Face2Gene: genetic identity behind deep facial features | NI | NI | Not found |
| Kim & Bodurtha | 2019 | Dysmorphology | Pediatrics in review | 10.1542/pir.2018-0331 PT - Article | Publication type |
| Lastivka et al. | 2022 | Diagnosis and verification of the autosomal dominant form of Kabuki makeup syndrome in a child with congenital heart disease. Clinical case | European journal of human genetics | NI | Not found |
| Latorre-Pellicer et al. | 2020 | Identifying Cornelia de Lange Syndrome by facial phenotypes using Face2Gene | European journal of human genetics | NI | Not found |
| Li et al. | 2023 | Increasing the usability of PEDIA approach by integrating open-source GestaltMatcher and CADA into variant prioritization pipeline | European journal of human genetics | 10.1038/s41431-023-01338-4 PT - Conference Abstract | Publication type |
| Lopergolo et al. | 2019 | Clinical application of a facial dysmorphology tool: a performance analysis | European journal of human genetics | 10.1038/s41431-018-0247-7 PT - Conference Abstract | Publication type |
| Lyon et al. | 2022 | eP182: KBG syndrome: prospective videoconferencing and use of AI-driven facial phenotyping | Genetics in medicine | 10.1016/j.gim.2022.01.218 PT - Conference Abstract | Publication type |
| Mak et al. | 2021 | Craniofacial features of 3q29 deletion syndrome: application of next-generation phenotyping technology | American journal of medical genetics part a | 10.1002/ajmg.a.62227 | Intervention |
| Martinez-Monseny et al. | 2019 | PMM2-CDG patients gestalt: is recognizable enough? | European journal of human genetics | NI | Not found |
| McCradden et al. | 2021 | The point-of-care use of a facial phenotyping tool in the genetics clinic: an ethics tete-a-tete | American journal of medical genetics part a | 10.1002/ajmg.a.61985 | Publication type |
| Mehr et al. | 2020 | Facial phenotyping in fabry disease using Face2Gene | Molecular genetics and metabolism | 10.1016/j.ymgme.2019.11.280 | Publication type |
| Morlan et al. | 2019 | The utility of computer-assisted facial recognition in the etiologic diagnosis of patients with global developmental delay & intellectual disability | European journal of human genetics | 10.1038/s41431-018-0247-7 PT - Conference Abstract | Publication type |
| Motokawa et al. | 2023 | Clinical usefulness of a deep learning-based facial recognition application (Face2Gene) as a diagnostic tool for syndromic endocrine-related disorders | Hormone research in paediatrics | NI | Not accessible |
| Myers | 2019 | Morphological variants in neurodevelopmental disorders | NI | NI | Publication type |
| Neves et al. | 2023 | KBG syndrome in the Portuguese population: clinical and molecular characterization of 41 patients | European journal of human genetics | NI | Not found |
| Pantel et al. | 2020 | Testing and improving DeepGestalt's discriminatory power on photographs of individuals with and without a genetic syndrome | European journal of human genetics | 10.1038/s41431-020-00739-z PT - Conference Abstract | Publication type |
| Perne et al. | 2023 | How DeepGestalt triggered WES re-analysis and led to the identification of a KANSL1 intragenic deletion causing Koolen-de Vries syndrome | European journal of human genetics | NI | Not found |
| Pi Castan et al. | 2024 | Team-based approach to using the Face2Gene platform - a Spanish experience | European journal of human genetics | NI | Not found |
| Porras et al. | 2021 | Development and evaluation of a machine learning-based point-of-care screening tool for genetic syndromes in children: a multinational retrospective study | Lancet digital health | 10.1016/S2589-7500(21)00137-0 | Intervention |
| Pratamawati et al. | 2017 | A family with three children of rare intellectual disability syndrome | Annals of translational medicine | 10.21037/atm.2017.s060 PT - Conference Abstract | Publication type |
| Ramos et al. | 2020 | Cornelia de Lange spectrum: does deep phenotyping help? | European journal of human genetics | NI | Not found |
| Randa et al. | 2019 | The importance of dysmorphology in genetic diagnosis-a case report with index finger anomaly | Erciyes medical journal | NI | Not found |
| Roalf et al. | 2022 | Computer-vision analysis of craniofacial dysmorphology in 22q11.2 deletion syndrome and psychosis spectrum disorders | Neuropsychopharmacology | 10.1038/s41386-022-01485-0 PT - Conference Abstract | Publication type |
| Roalf et al. | 2021 | Using next-generation facial analysis to elucidate neurodevelopmental patterns associated with craniofacial abnormalities in 22q11.2 deletion syndrome and in patients along the psychosis-spectrum | Neuropsychopharmacology | NI | Not found |
| Schmidt et al. | 2019 | Pitt-Hopkins syndrome identified by trio whole-exome sequencing in a boy with severe intellectual disability, postnatal microcephaly, myopia, scoliosis and congenital abnormalities of the elbow joint | European journal of human genetics | 10.1038/s41431-018-0247-7 PT - Conference Abstract | Publication type |
| Shi et al. | 2018 | Integrated facial analysis and targeted sequencing identifies a novel KDM6A pathogenic variant resulting in Kabuki syndrome | Journal of bio-X research | 10.1097/JBR.0000000000000022 PT - Article | Not found |
| Tekendo-Ngongang & Kruszka | 2020 | Noonan syndrome on the African Continent | Birth defects research | 10.1002/bdr2.1675 | Publication type |
| Tordjman et al. | 2018 | Interest of searching dysmorphic features in autism spectrum disorder: comparison of clinical geneticists and Face2Gene photos analyses | European journal of human genetics | NI | Not found |
| Valverde de Morales et al. | 2023 | Expansion of the genotypic and phenotypic spectrum of CTCF-related disorder guides clinical management: 43 new subjects and a comprehensive literature review | American journal of medical genetics part a | 10.1002/ajmg.a.63065 | Intervention |
| Van De Lande | 2022 | 3d statistical shape analysis of the face in apert syndrome | NI | NI | Not found |
| Vuocolo et al. | 2024 | Improving access to exome sequencing in a medically underserved population through the texome project | Genetics in medicine | 10.1016/j.gim.2024.101102 | Intervention |
| Wang et al. | 2019 | The phenotypic spectrum of kabuki syndrome in patients of chinese descent | Hormone research in paediatrics | 10.1159/000501868 PT - Conference Abstract | Publication type |

# Details on included studies

## Key Characteristics of Included Studies

| **Study** | **Country** | **Design** | **Condition** | **Face2Gene-related Outcomes** | **Cases in Face2Gene Analysis** |
| --- | --- | --- | --- | --- | --- |
| Arora et al. [1] | India | Single case study | Wiedemann-Steiner syndrome | Case description | 1 |
| Arora et al. [2] | India | Single case study | Osteopathia striata with cranial sclerosis | Case description | 1 |
| Bezuidenhout et al. [3] | South Africa | Multi case study | Hyperphosphatasia with mental retardation syndrome type 4 | Case description | 3 |
| Carli et al. [4] | Italy | Case-control study | Pathogenic variants in neuroblastoma‐amplified sequence | Condition description | 32 |
| Carrer et al. [5] | Italy | Retrospective validation | Multiple | Top-3, Top-10, Top-30 accuracy | 145 |
| Ciancia et al. [6] | Netherlands | Retrospective validation | Silver-Russell syndrome and Prader-Willi syndrome | Top-1, Top-5, Top-10 sensitivity | 52 |
| Echeverry-Quiceno et al. [7] | Colombia, Spain | Case-control study | Multiple | Facial landmarks, Top-1, Top-5 accuracy | 130 |
| Elmas & Gogus [8] | Turkey | Retrospective validation | Multiple | Case description, gestalt score, feature score, combined score, rank at suggested syndrome list | 25 |
| Elmas & Gogus [9] | Turkey | Retrospective validation | Neurofibromatosis type 1 | Presence and sequence of the syndrome diagnosed, gestalt score, feature score | 56 |
| Garcia et al. [10] | USA | Single case study | Cornelia de Lange syndrome | Case description, facial heatmap | 1 |
| Gnazzo et al. [11] | Italy | Single case study | Usmani-Riazuddin syndrome | Case description, possible diagnosis provided by F2G | 1 |
| Guo et al. [12] | USA | Multi case study | KBG syndrome | Condition description. Top-10 and Top-30 ranks, Top-1, Top-10, Top-30 accuracy | 25 |
| Kim SK et al. [13] | South Korea | Retrospective validation | Multiple | Top-10 accuracy, gestalt similarity, diagnostic yield | 23 |
| Kim JS et al. [14] | South Korea | Single case study | Noonan syndrome | Case description | 1 |
| Knaus et al. [15] | Germany | Multi case study | Multiple | True positive and false negative rates, mean accuracy | 91 |
| Kusikova et al. [16] | Austria, Slovakia | Retrospective validation | X-linked myotubular myopathy | Presence and sequence of the syndrome diagnosed, AUC | 56 |
| Latorre-Pellicer et al. [44] | Spain | Retrospective validation | Cornelia de Lange syndrome | Top-1, Top-5 ranks, evaluation for different ages, genetic variants | 49 |
| Liehr et al. [17] | Germany | Case-control study | Emanuel & Pallister-Killian syndrome | Top-1, Top-5 ranks, AUC | 2075 |
| Lubala et al. [18] | DR Congo | Retrospective validation | Fragile X syndrome | Mean accuracy, AUC, true positive, false negative rate, heatmaps | 156 |
| Lumaka et al. [19] | DR Congo | Retrospective validation & algorithm training | Down syndrome | Top-10 ranks, mean rank before and after training | 127 |
| Martinez-Monseny et al. [20] | Spain | Case-control study + model training | Phosphomannomutase-2 deficiency | Condition description, Top-10 ranks, AUC | 31 |
| Marwaha et al. [21] | Canada | Multi case study | Kabuki syndrome & CDK13-related disorder | Case descriptions, gestalt score | 2 |
| Marwaha et al. [22] | Canada | Prospective evaluation | Multiple | Top-10 accuracy, diagnostic yield, provider experience | 72 |
| Mishima et al. [23] | Japan | Prospective evaluation | Congenital dysmorphic syndromes & Down syndrome | Top-1, Top-3, Top-10 sensitivity, evaluation for different ages | 108 |
| Mubungu et al. [24] | n.s. | Single case study | Xia-Gibbs syndrome | Case description, top match | 1 |
| Myers et al. [25] | Sweden | Case-control study | Neurodevelopmental disorders | Identification of facial morphological variants, agreement between F2G results and clinical assessment, ROC analysis | 1 |
| Narayanan et al. [26] | India | Retrospective validation | Multiple | Case description, Top-1, Top-10 ranks | 51 |
| Obara et al. [27] | Japan | Single case study | Intellectual developmental disorder, autosomal dominant 7 | Case description | 1 |
| Pantel et al. [28] | Germany | Case-control study | Multiple | Top-10 sensitivity, AUROC, results for different ethnic groups | 323 |
| Park et al. [29] | South Korea | Multi case study | Tatton-Brown-Rahman syndrome & Say-Barber-Biesecker-Young-Simpson ​variant of ohdo syndrome | Case description, Top-10 results | 2 |
| Pascolini et al. [30] | Italy | Single case study | White-Sutton syndrome | Case description | 1 |
| Pascolini et al. [31] | Italy | Retrospective validation + Case study | Nicolaides-Baraitser & Coffin-Siris syndroms | Case description, identification of the diagnosis | 20 |
| Pascolini et al. [32] | Italy | Case-control study | Trichothiodystrophies | Condition description, AUC | 38 |
| Pascolini et al. [33] | Italy | Retrospective validation | Multiple | Top-1, Top-10, Top-30 ranks | 19 |
| Pode-Shakked et al. [34] | Israel | Case-control study | Mucolipidosis type IV | AUC | 207 |
| Reiter et al. [35] | Germany | Case-control study | Multiple | Top-1, Top-10, Top-30 sensitivity, AUROC, D-score | 5119 |
| Srisraluang & Rojnueangnit [36] | Thailand | Prospective evaluation | Down syndrome | Sensitivity, specificity, accuracy, PPV, NPV, total correct score | 64 |
| Tripon et al. [37] | Romania | Single case study | Pitt-Hopkins syndrome | Case description | 1 |
| Vorravanpreecha et al. [38] | Thailand | Case-control study | Down syndrome | Sensitivity, accuracy, specificity, AUC, Top-9 matches besides Down syndrome | 170 |
| Zhang et al. [39] | China | Case-control study | Alström syndrome | Facial landmarks, AUC | 66 |
| **Notes.** Abbreviations: n.s. Not specified, AUC Area under the curve, ROC Receiver operating characteristic, PPV Positive predictive value, NPV negative predictive value, AUROC Area under the receiver operating characteristic curve | | | | | |

## References of Included Studies

[1] Arora V, Puri RD, Bijarnia-Mahay S, Verma IC. Expanding the phenotypic and genotypic spectrum of Wiedemann-Steiner syndrome: First patient from India. American Journal of Medical Genetics Part A 2020;182:953–6. https://doi.org/10.1002/ajmg.a.61534.

[2] Arora V, Bijarnia-Mahay S, Saxena KK, Suman P, Kukreja S. Osteopathia Striata with Cranial Sclerosis: A Face-to-Radiograph-to-Gene Diagnosis. Journal of Pediatric Genetics 2022;11:63–7. https://doi.org/10.1055/s-0040-1715120.

[3] Bezuidenhout H, Bayley S, Smit L, Kinnear C, Moller M, Uren C, et al. Hyperphosphatasia with mental retardation syndrome type 4 in three unrelated South African patients. American Journal of Medical Genetics Part A 2020;182:2230–5. https://doi.org/10.1002/ajmg.a.61797.

[4] Carli D, Giorgio E, Pantaleoni F, Bruselles A, Barresi S, Riberi E, et al. *NBAS* pathogenic variants: Defining the associated clinical and facial phenotype and genotype-phenotype correlations. Human Mutation 2019;40:721–8. https://doi.org/10.1002/humu.23734.

[5] Carrer A, Romaniello MG, Calderara ML, Mariani M, Biondi A, Selicorni A. Application of the Face2Gene tool in an Italian dysmorphological pediatric clinic: Retrospective validation and future perspectives. American Journal of Medical Genetics Part A 2024;194. https://doi.org/10.1002/ajmg.a.63459.

[6] Ciancia S, Goedegebuure WJ, Grootjen LNN, Hokken-Koelega ACS, Kerkhof GFF, van der Kaay DCM. Computer-aided facial analysis as a tool to identify patients with Silver-Russell syndrome and Prader-Willi syndrome. European Journal of Pediatrics 2023;182:2607–14. https://doi.org/10.1007/s00431-023-04937-x.

[7] Echeverry-Quiceno LMM, Candelo E, Gomez E, Solis P, Ramirez D, Ortiz D, et al. Population-specific facial traits and diagnosis accuracy of genetic and rare diseases in an admixed Colombian population. Scientific Reports 2023;13. https://doi.org/10.1038/s41598-023-33374-x.

[8] Elmas M, Gogus B. Success of Face Analysis Technology in Rare Genetic Diseases Diagnosed by Whole-Exome Sequencing: A Single-Center Experience. Molecular Syndromology 2020;11:4–14. https://doi.org/10.1159/000505800.

[9] Elmas M, Gogus B. The road from mutation to next generation phenotyping: contribution of deep learning technology (Face2Gene) to diagnosis neurofibromatosis type 1. European Research Journal 2022;8:145–54. https://dx.doi.org/10.18621/eurj.894631 PT - Article.

[10] Garcia AG, Malone J, Li H. A novel mosaic variant on *SMC1A* reported in buccal mucosa cells, albeit not in blood, of a patient with Cornelia de Lange-like presentation. Cold Spring Harbor Molecular Case Studies 2020;6. https://doi.org/10.1101/mcs.a005322.

[11] Gnazzo M, Pascolini G, Parlapiano G, Petrizzelli F, Perrino D, Porco L, et al. Usmani-Riazuddin syndrome can have a recognizable phenotype: Report of a novel *AP1G1* variant. Clinical Genetics 2024. https://doi.org/10.1111/cge.14531.

[12] Guo L, Park J, Yi E, Marchi E, Hsieh T-C, Kibalnyk Y, et al. KBG syndrome: videoconferencing and use of artificial intelligence driven facial phenotyping in 25 new patients. European Journal of Human Genetics 2022;30:1244–54. https://doi.org/10.1038/s41431-022-01171-1.

[13] Kim H, Kang SW, Kim J-H, Nagar H, Sabuncu M, Margolis DJA, et al. The role of AI in prostate MRI quality and interpretation: Opportunities and challenges. European Journal of Radiology 2023;165:110887. https://doi.org/10.1016/j.ejrad.2023.110887.

[14] Kim SK, Yoon JS, phil B seong, Kim J, Jeongho L, Lee DH. A case of Noonan syndrome diagnosed using the facial recognition software (FACE2GENE). Journal of Genetic Medicine 2019;16:81–4. https://doi.org/10.5734/JGM.2019.16.2.81.

[15] Knaus A, Pantel JT, Pendziwiat M, Hajjir N, Zhao M, Hsieh T-C, et al. Characterization of glycosylphosphatidylinositol biosynthesis defects by clinical features, flow cytometry, and automated image analysis. Genome Med 2018;10:3. https://doi.org/10.1186/s13073-017-0510-5.

[16] Kusikova K, Soltysova A, Ficek A, Feichtinger RG, Mayr JA, Skopkova M, et al. Prognostic Value of Genotype-Phenotype Correlations in X-Linked Myotubular Myopathy and the Use of the Face2Gene Application as an Effective Non-Invasive Diagnostic Tool. Genes 2023;14. https://doi.org/10.3390/genes14122174.

[17] Liehr T, Acquarola N, Pyle K, St-Pierre S, Rinholm M, Bar O, et al. Next generation phenotyping in Emanuel and Pallister-Killian syndrome using computer-aided facial dysmorphology analysis of 2D photos. Clinical Genetics 2018;93:378–81. https://doi.org/10.1111/cge.13087.

[18] Lubala TK, Kayembe-Kitenge T, Mubungu G, Lumaka A, Kanteng G, Savage S, et al. Usefulness of automated image analysis for recognition of the fragile X syndrome gestalt in Congolese subjects. European Journal of Medical Genetics 2023;66. https://doi.org/10.1016/j.ejmg.2023.104819.

[19] Lumaka A, Cosemans N, Mampasi AL, Mubungu G, Mvuama N, Lubala T, et al. Facial dysmorphism is influenced by ethnic background of the patient and of the evaluator. Clinical Genetics 2017;92:166–71. https://doi.org/10.1111/cge.12948.

[20] Martinez-Monseny A, Cuadras D, Bolasell M, Muchart J, Arjona C, Borregan M, et al. From gestalt to gene: early predictive dysmorphic features of PMM2-CDG. Journal of Medical Genetics 2019;56:236–45. https://doi.org/10.1136/jmedgenet-2018-105588.

[21] Marwaha A, Costain G, Cytrynbaum C, Mendoza-Londano R, Chad L, Awamleh Z, et al. The utility of DNA methylation signatures in directing genome sequencing workflow: Kabuki syndrome and CDK13-related disorder. American Journal of Medical Genetics Part A 2022;188:1368–75. https://doi.org/10.1002/ajmg.a.62650.

[22] Marwaha A, Chitayat D, Meyn M, Mendoza-Londono R, Chad L. The point-of-care use of a facial phenotyping tool in the genetics clinic: Enhancing diagnosis and education with machine learning. American Journal of Medical Genetics Part A 2021;185:1151–8.

[23] Mishima H, Suzuki H, Doi M, Miyazaki M, Watanabe S, Matsumoto T, et al. Evaluation of Face2Gene using facial images of patients with congenital dysmorphic syndromes recruited in Japan. J Hum Genet 2019;64:789–94. https://doi.org/10.1038/s10038-019-0619-z.

[24] Mubungu G, Makay P, Boujemla B, Yanda S, Posey JE, Lupski JR, et al. Clinical presentation and evolution of Xia-Gibbs syndrome due to p.Gly375ArgfsTer3 variant in a patient from DR Congo (Central Africa). American Journal of Medical Genetics Part A 2021;185:990–4. https://doi.org/10.1002/ajmg.a.62049.

[25] Myers L, Anderlid B-M, Nordgren A, Lundin K, Kuja-Halkola R, Tammimies K, et al. Clinical versus automated assessments of morphological variants in twins with and without neurodevelopmental disorders. American Journal of Medical Genetics Part A 2020;182:1177–89. https://doi.org/10.1002/ajmg.a.61545.

[26] Narayanan DL, Ranganath P, Aggarwal S, Dalal A, Phadke SR, Mandal K. Computer-aided Facial Analysis in Diagnosing Dysmorphic Syndromes in Indian Children. Indian Pediatr 2019;56:1017–9.

[27] Obara K, Abe E, Toyoshima I. Whole-Exome Sequencing Identified a Novel DYRK1A Variant in a Patient With Intellectual Developmental Disorder, Autosomal Dominant 7. Cureus Journal of Medical Science 2023;15. https://doi.org/10.7759/cureus.33379.

[28] Pantel JT, Hajjir N, Danyel M, Elsner J, Abad-Perez AT, Hansen P, et al. Efficiency of Computer-Aided Facial Phenotyping (DeepGestalt) in Individuals With and Without a Genetic Syndrome: Diagnostic Accuracy Study. J Med Internet Res 2020;22:e19263. https://doi.org/10.2196/19263.

[29] Park S, Kim J, Song T-Y, Jang D-H. Case Report: The success of face analysis technology in extremely rare genetic diseases in Korea: Tatton-Brown-Rahman syndrome and Say-Barber -Biesecker-Young-Simpson variant of ohdo syndrome. Frontiers in Genetics 2022;13. https://doi.org/10.3389/fgene.2022.903199.

[30] Pascolini G, Agolini E, Fleischer N, Gulotta E, Cesario C, D’Elia G, et al. A novel patient with White-Sutton syndrome refines the mutational and clinical repertoire of the *POGZ-*related phenotype and suggests further observations. American Journal of Medical Genetics Part A 2020;182:1791–5. https://doi.org/10.1002/ajmg.a.61605.

[31] Pascolini G, Calvani M, Grammatico P. First Italian experience using the automated craniofacial gestalt analysis on a cohort of pediatric patients with multiple anomaly syndromes. Italian Journal of Pediatrics 2022;48. https://doi.org/10.1186/s13052-022-01283-w.

[32] Pascolini G, Gaudioso F, Baldi M, Alario D, Dituri F, Novelli A, et al. Facial clues to the photosensitive trichothiodystrophy phenotype in childhood. Journal of Human Genetics 2023;68:437–43. https://doi.org/10.1038/s10038-023-01134-4.

[33] Pascolini G, Valiante M, Bottillo I, Laino L, Fleischer N, Ferraris A, et al. Striking phenotypic overlap between Nicolaides-Baraitser and Coffin-Siris syndromes in monozygotic twins with *ARID1B* intragenic deletion. European Journal of Medical Genetics 2020;63. https://doi.org/10.1016/j.ejmg.2019.103739.

[34] Pode-Shakked B, Finezilber Y, Levi Y, Liber S, Fleischer N, Greenbaum L, et al. Shared facial phenotype of patients with mucolipidosis type IV: A clinical observation reaffirmed by next generation phenotyping. European Journal of Medical Genetics 2020;63. https://doi.org/10.1016/j.ejmg.2020.103927.

[35] Reiter AMV, Pantel JT, Danyel M, Horn D, Ott C-E, Mensah MA. Validation of 3 Computer-Aided Facial Phenotyping Tools (DeepGestalt, GestaltMatcher, and D-Score): Comparative Diagnostic Accuracy Study. J Med Internet Res 2024;26:e42904. https://doi.org/10.2196/42904.

[36] Srisraluang W, Rojnueangnit K. Facial recognition accuracy in photographs of Thai neonates with Down syndrome among physicians and the Face2Gene application. American Journal of Medical Genetics Part A 2021;185:3701–5. https://doi.org/10.1002/ajmg.a.62432.

[37] Tripon F, Boglis A, Micheu C, Streata I, Banescu C. Pitt-Hopkins Syndrome: Clinical and Molecular Findings of a 5-Year-Old Patient. Genes 2020;11. https://doi.org/10.3390/genes11060596.

[38] Vorravanpreecha N, Lertboonnum T, Rodjanadit R, Sriplienchan P, Rojnueangnit K. Studying Down syndrome recognition probabilities in Thai children with de-identified computer-aided facial analysis. American Journal of Medical Genetics Part A 2018;176:1935–40. https://doi.org/10.1002/ajmg.a.40483.

[39] Zhang Y, Shi J, Peng Y, Zhao Z, Zheng Q, Wang Z, et al. Artificial intelligence-enabled screening for diabetic retinopathy: a real-world, multicenter and prospective study. BMJ Open Diab Res Care 2020;8:e001596. https://doi.org/10.1136/bmjdrc-2020-001596.

# Perceived benefits of AI use in Genetics

| **Benefit** | **Definition** | **Number of Codes** | **Number of Interviews** | **Example** |
| --- | --- | --- | --- | --- |
| Process support | AI supports clinicians in the work process, for example by recognizing and analysing more complex data patterns. | 15 | 10 | "[…] AI will be able to recognize structures, bring in structures that you would otherwise have to do manually, where it would take years or never be possible." (IV 14) |
| Increased efficiency | Acceleration or optimization of the work process through AI. | 9 | 8 | "I believe that you could actually shorten the time to diagnosis with many of these tools." (IV 06) |
| Improved diagnostics | Use of AI improves the safety and quality of diagnosis. | 11 | 7 | "I believe that we absolutely need to use artificial intelligence because the range of diagnoses is increasing. There are more and more diagnoses that you can no longer know […]. And I definitely see the need for AI as a support system here." (IV 09) |
| Reduce burden of staff | AI relieves staff by automating or taking over tasks. | 5 | 4 | "And I believe that it will be more of a positive influence because it can simply be a support in order to be able to cope with all the workload." (IV 17) |

# Perceived RISKS of AI use in Genetics

| **Risk** | **Definition** | **Number of Codes** | **Number of Interviews** | **Example** |
| --- | --- | --- | --- | --- |
| Misuse | AI or the data uploaded is not used for its intended purpose potentially causing harm to people. | 9 | 9 | "[…] one risk is data protection. That sensitive data, especially genetic data, which I somehow still consider to be more sensitive than other data, could fall into the wrong hands." (IV 05) |
| Overtrust | Risk that clinicians do not critically evaluate AI results and rely the results for their diagnosis, with the possible risk of false diagnosis when AI is incorrect. | 9 | 8 | "I'm a bit critical of the use when people […] solely rely on what the AI comes up with." (IV 08) |
| Bias | Risk of a potential bias in AI output due to certain patient groups being under- or overrepresented in the training dataset of the AI. | 3 | 3 | "And since there is more of a risk […] that the AI itself will get bias from the training set etc. And then perhaps performs differently for different patient groups and this is not understood." (IV 09) |
| Loss of competencies | Risk of clinicians losing their skills due to frequent AI use or clinicians in-training do not acquire skills due to learning with AI assistance only. | 3 | 2 | "So all the simplifications […] brought about by technical innovations always automatically lead to things being handed over. In other words, you give up medical activities and lose skills." (IV 14) |
